# Robust Rank Aggregation and Least Absolute Shrinkage and Selection Operator Analysis of Novel Gene Signatures in Dilated Cardiomyopathy

**DOI:** 10.3389/fcvm.2021.747803

**Published:** 2021-12-14

**Authors:** Xiao Ma, Changhua Mo, Liangzhao Huang, Peidong Cao, Louyi Shen, Chun Gui

**Affiliations:** Department of Cardiology, First Affiliated Hospital of Guangxi Medical University, Nanning, China

**Keywords:** dilated cardiomyopathy, Robust Rank Aggregation, novel gene signatures, Least absolute shrinkage and selection operator analysis, PERELP

## Abstract

**Objective:** Dilated cardiomyopathy (DCM) is a heart disease with high mortality characterized by progressive cardiac dilation and myocardial contractility reduction. The molecular signature of dilated cardiomyopathy remains to be defined. Hence, seeking potential biomarkers and therapeutic of DCM is urgent and necessary.

**Methods:** In this study, we utilized the Robust Rank Aggregation (RRA) method to integrate four eligible DCM microarray datasets from the GEO and identified a set of significant differentially expressed genes (DEGs) between dilated cardiomyopathy and non-heart failure. Moreover, LASSO analysis was carried out to clarify the diagnostic and DCM clinical features of these genes and identify dilated cardiomyopathy derived diagnostic signatures (DCMDDS).

**Results:** A total of 117 DEGs were identified across the four microarrays. Furthermore, GO analysis demonstrated that these DEGs were mainly enriched in the regulation of inflammatory response, the humoral immune response, the regulation of blood pressure and collagen–containing extracellular matrix. In addition, KEGG analysis revealed that DEGs were mainly enriched in diverse infected signaling pathways. Moreover, Gene set enrichment analysis revealed that immune and inflammatory biological processes such as adaptive immune response, cellular response to interferon and cardiac muscle contraction, dilated cardiomyopathy are significantly enriched in DCM. Moreover, Least absolute shrinkage and selection operator (LASSO) analyses of the 18 DCM-related genes developed a 7-gene signature predictive of DCM. This signature included ANKRD1, COL1A1, MYH6, PERELP, PRKACA, CDKN1A, and OMD. Interestingly, five of these seven genes have a correlation with left ventricular ejection fraction (LVEF) in DCM patients.

**Conclusion:** Our present study demonstrated that the signatures could be robust tools for predicting DCM in clinical practice. And may also be potential treatment targets for clinical implication in the future.

## Introduction

Dilated cardiomyopathy (DCM), characterized by left ventricular or bicentricular enlargement and myocardial systolic dysfunction, is the main cause of systolic heart failure and heart transplantation in about 40 million people worldwide (1, 2). The etiology of dilated cardiomyopathy is still not clear, and it is generally believed that people with genetic background of dilated cardiomyopathy are usually infected with Coxsackie virus, adenovirus and influenza virus, and then resulting cardiac inflammation and immune damage jointly lead to the occurrence and development of dilated cardiomyopathy (3, 4). In recent decades, many strategies including early diagnosis, more accurate typing, evidence-based treatment, rigorous follow-up, and the use of advanced anti-heart failure drugs, have improved the quality of life and long-term survival of patients with dilated cardiomyopathy (5, 6). However, patients with dilated cardiomyopathy presenting with symptoms of heart failure have a poor clinical prognosis, with a 5-year mortality rate of ~20% in these patients (7, 8). At present, the pathogenesis of dilated cardiomyopathy is still poorly understood.

Advances in gene chips and high-throughput sequencing help to identify the key role of potential core genes and small molecule in the biological process of a variety of diseases (9, 10). Many studies have used microarray chip technology to measure the gene and non-coding RNA expression profile of dilated cardiomyopathy, providing the gene expression pattern of the myocardial tissue of dilated cardiomyopathy. For example, Zhang et al. used bioinformatics method to reanalyze gene expression profile and potential functional network in cardiac tissue of patients with dilated cardiomyopathy (11). Tao et al. also reported four hub lncRNAs in the DCM-related module, which helped to enhance the understanding of DCM pathophysiological process and reveal its potential treatment targets (12). However, most of the gene signatures were analyzed from a single data set, and a limited number of patients have been included in most previous studies, which may compromise the prediction power or reliability. In-depth exploration of the public datasets can reveal disease related genes and develop a efficient risk gene signature in combination with clinicopathological characteristics (13), which can help to form a promising tool for predicting status of DCM and individualized therapy.

To explore potential pathogenesis and therapeutic targets of DCM, a series of analysis based on microarray chip data were performed. we developed a 7-gene DCM derived diagnosis signature (DCMDDS) distinguished DCM from NC with high specificity and sensitivity in both the training and validation cohorts. Besides, we performed gene-clinical feature correlation analysis on the 7-gene DCMDS, and predicted potential therapeutic targets via the DGIdb database. The present study provided new diagnostic markers and potential gene-based targeted treatment drug for DCM.

## Methods

### Data Download

**Figure 1:**
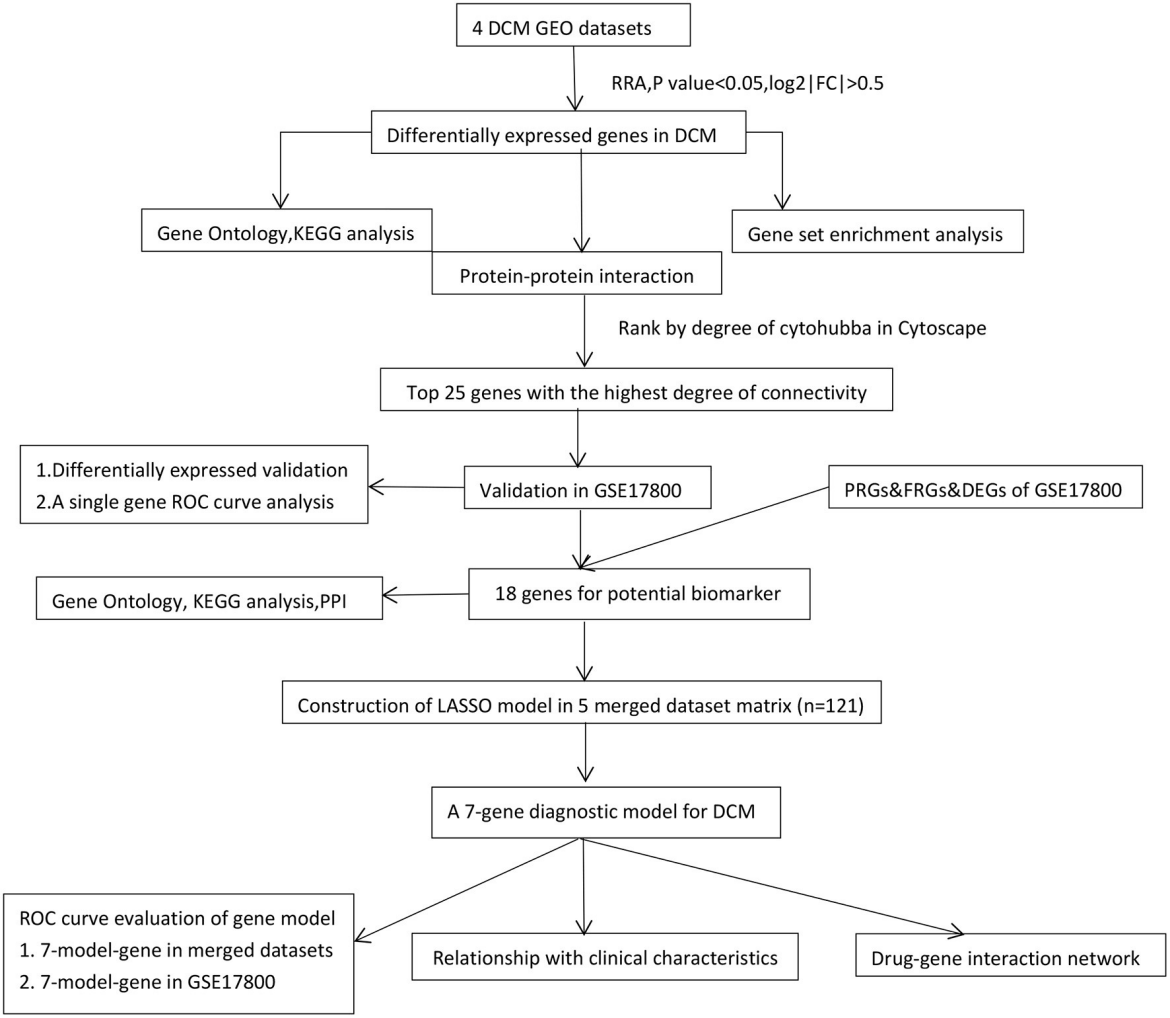
The workflow of the study. DCM, dilated cardiomyopathy; RRA, Robust Rank Aggregation; LASSO, Least absolute shrinkage and selection operator; DEGs, differentially expressed genes; KEGG, Kyoto Encyclopedia of Genes and Genomes; PPI, protein-protein interaction; Con, control normal cardiac tissues; ROC, receiver operating characteristic; FRGs, ferroptosis-related genes; PRGs, pyroptosis-related genes.

The workflow of the present study was shown in Figure 1. Four gene expression profiles of DCM, including GSE3585, GSE9800, GSE21610, and GSE42955, were downloaded from the Gene Expression Omnibus (GEO) (http://www.ncbi.nlm.nih.gov/geo accessed on 17 June 2021) and used to identify DEGs between DCM and normal cardiac (NC) tissue samples (Table 1). The criteria for selecting these datasets included: (1) Gene expression data must be available for both DCM and NCT samples, (2) at least 5,000 genes must be included when the microarray platform is used for expression profiling. In general, DCM is defined by patients with clinical features of a left ventricular end-diastolic diameter over than 56 mm and a left ventricular ejection fraction (LVEF) <50% (14). Exclusion criteria were genetic DCM or any cardiovascular, life-limiting systemic condition or an infectious or tumoral condition that may influence the definition of DCM. Of the five datasets (including the GSE17800 dataset below) with DCM, only four provided DCM definition (GSE3585 GSE17800, GSE21610 and GSE42955), and both datasets used the above criteria. In the GSE9800, a patient's DCM status was provided without specifying how it was defined. GEO belongs to public databases. The patients involved in the database have obtained ethical approval. Users can download these relevant data for free for research and publish relevant articles. A majority of data are based on open source data, so there are no ethical issues and other conflicts of interest.

**Table 1:** The characteristic baseline of microarray.

**Series accession**	**Normal(n)**	**DCM(n)**	**Samples**	**Platform**	**Author ref**	**Application**
GSE3585	5	7	left ventricular tissue samples	GPL96 Affymetrix Human Genome U133A Array	Barth AS et al.	Integrated Analysis
GSE42955	5	12	left ventricle tissue samples	GPL6244 Affymetrix Human Gene 1.0 ST Array	Molina-Navarro MM et al.	Integrated Analysis
GSE9800	2	12	Left ventricular tissue samples	GPL887 Agilent-012097 Human 1A Microarray G4110B	Ohtsuki M et al.	Integrated Analysis
GSE21610	8	22	Left ventricular tissue samples	GPL570 [HG-U133_Plus_2] Affymetrix Human Genome U133 Plus 2.0 Array	Patrick Schwientek. et al.	Integrated Analysis
GSE17800	8	40	Myocardial biopsies tissue samples	GPL570 [HG-U133_Plus_2] Affymetrix Human Genome U133 Plus 2.0 Array	Sabine Ameling et al.	Validation and clinical relevance analysis

### Data Preprocessing and Identification of Robust Differentially Expressed Genes (DEGs)

R software (version 3.6.1) was performed to process and statistically analyze the expression files. We downloaded the series matrix files of datasets from GEO. The R package “limma” (15) was utilized to normalize the data and find DEGs, and a volcano map of DEGs was drawn using the “ggplot2” package (16) to show the DEGs. We then used RRA to integrate the results of those 4 datasets to find the most significant DEGs (17). The *P* value of each gene indicated its ranking in the final gene list, and genes with adjusted *P* < 0.05 and log2|FC| > 0.5 were considered as significant DEGs in the RRA analysis.

### Enrichment Analyses of GO and KEGG Pathway

Gene Ontology (GO) analysis and Kyoto Encyclopedia of Genes and Genomes (KEGG) analysis of DEGs were calculated using clusterProfiler package. clusterProfiler was a package of R software that was designed to compare and visualize functional profiles among gene clusters (18). A *P* value < 0.05 was considered to be significant, and the identified significant analyses were sorted by gene counts. Subsequently, the R package “RCircos” (version 1.2.1) (19) was used to visualize the expression patterns of different microarrays and chromosomal locations for the top 40 DEGs sorted by their *P* value.

### Gene Set Enrichment Analysis

We performed gene set enrichment analysis (GSEA) using the gene expression matrix through the “clusterProfiler” package. “c2.cp.kegg.v7.0.symbols.gmt” was selected as the reference gene set (20). A false discovery rate (FDR) < 0.25 and *P* < 0.05 were considered significant enrichment.

### Protein-Protein Interaction (PPI) Network

The PPI network was constructed with a threshold of medium confidence ≥0.4 through the Search Tool for the Retrieval of Interacting Genes (STRING) database (21). Cytoscape software (v3.6.1; http://www.cytoscape.org/) was used to visualize the network. Then, the top 25 genes with highest connectivity in the network were identified by DEGREE in cytoHubba (22).

### The Identification and Validation of Hub Genes

The mRNA levels of high connectivity genes were verified in datasets GSE17800 and Student's *t* test was used to compare the expression levels of DCM and control groups. A list of 259 ferroptosis-related genes (FRGs) was identified through the ferroptosis database (FerrDb; http://www.zhounan.org/ferrdb) (23), a publicly available database of ferroptosis regulators, markers, and disease associations. A list of 34 pyroptosis-related genes (PRGs) from prior reviews and studies were also extracted (24, 25). The DEGs of GSE17800 was performed by limma package. And then, the DEGs in GSE17800 were intersected with FRGs/PRGs to obtain potential hub genes related to ferroptosis or pyroptosis. The high connectivity genes after validation and hub FRGs/PRGs were imported into the NCBI website for evaluating the expression abundance in normal human cardiac via high-throughput sequencing. Then, the gene expression with a threshold of RPKM in cardiac tissue ≥5 in NCBI Gene expression column was regarded as hub gene. To evaluate the identified ability of hub genes in DCM, ROC curve analysis were conducted in the GSE17800 data set through pROC package (26).

### Construction of a Diagnostic Model and Correlation Analysis for DCM

To investigate whether the hub genes could be applied for predicting DCM occurrence, five datasets from the GEO database, GSE3585, GSE42955, GSE9800, GSE21610 and GSE17800, were pooled together, and the combined dataset was then adjusted for batch effect through the “ComBat” function of sva (version 3.34.0) R package (27) and assigned as the training set. The transcriptional profile of GSE17800 which included 40 DCM and 8 no-heart failure (NHF) samples, was used for the validation of the model. The predictability of the model was then evaluated by area under the curve (AUC) of ROC. The “ggstatsplot” package (https://indrajeetpatil.github.io/ggstatsplot/) was used to perform Spearman correlation analysis on diagnostic markers and the “ggplot2” package was used to visualize the results. A two-sided *p* < 0.05 was considered to be statistically significant.

## Results

### Integrated Screening for Robust DCM-Associated Genes

**Figure 2:**
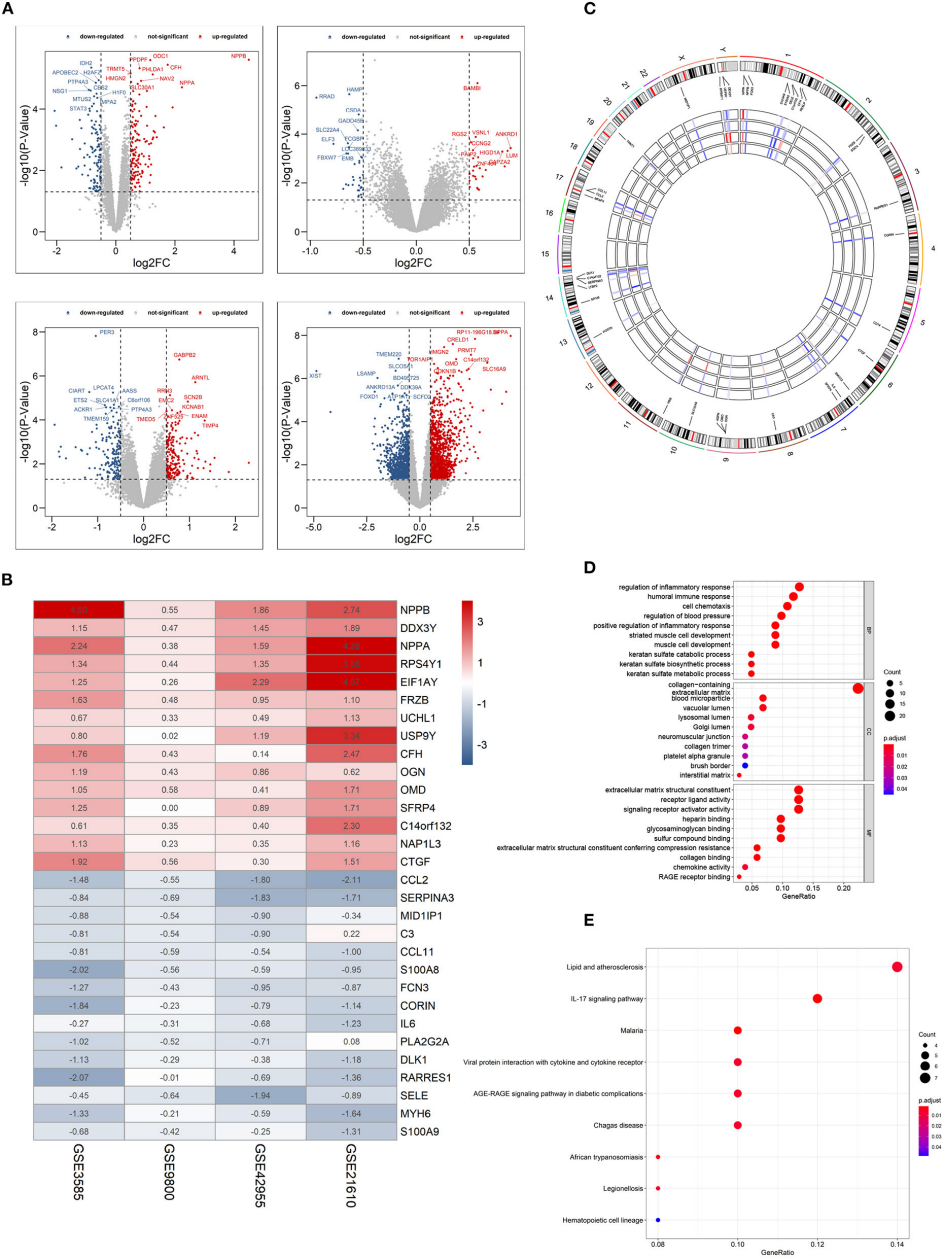
Identification of differentially expressed genes (DEGs) between dilated cardiomyopathy cardiac and normal cardiac tissues samples from four unrelated cohorts. **(A)** Volcano plots of datasets GSE3585 and GSE9800 (upper panel), and GSE21610 and GSE42955 (lower panel) from the GEO database. DEGs with |log2 (Fold change)| > 0.5 would be listed, additionally. **(B)** Heatmap of the top 15 downregulated and the top 15 upregulated genes from Robust Rank Aggregation analysis of four datasets. Each column represents a dataset and each row a gene. The number in each rectangle represents the value of log2 (fold change). The gradual color ranging from blue to red represents the changing process from down- to up-regulation (DCM vs. Control). **(C)** Circular visualization of expression patterns and chromosomal positions of the top 40 DEGs. The outer circle represents chromosomes, and lines coming from each gene point to their specific chromosomal locations. **(D)** GO enrichment analyses of differentially expressed genes (DEGs) between localized DCM and normal cardiac samples. **(E)** KEGG pathway analyses of differentially expressed genes (DEGs) between localized DCM and normal cardiac samples. DCM, dilated cardiomyopathy; GO, Gene Ontology; KEGG, Kyoto Encyclopedia of Genes and Genomes. The size of each dot represents the count of genes, and the color represents the *p*-value.

Four GEO datasets were used for the identification of robust DCM-associated genes (Table 1). Using the “limma” R package, we normalized expression data from datasets GSE3585, GSE9800, GSE21610, and GSE42955, and identified 253, 60, 2078, and 370 DEGs between DCM and normal cardiac tissues respectively, with a cut-off of *p* < 0.05 and log2|FC| > 0.5 (Figure 2A). Integration of all genes by the RRA method resulted in 117 DEGs (|log2FC|≥0.5, *p* < 0.05), 100 of which were upregulated and 17 downregulated in DCM.

The top 15 upregulated and the top 15 downregulated genes in CRPC are shown as hierarchical cluster heatmaps (Figure 2B). For the top 40 DEGs between DCM and normal cardiac tissues, their expression patterns across the four datasets used for analysis, along with their chromosomal locations are shown in a circos plot (Figure 2C). The location of these 40 DEGs involves almost all chromosomes except for the 12, 15, 16, 18, 20, 21, 22 chromosome. The top three upregulated genes included NPPB, NPPA, and EIF1AY, and they are located on chromosomes 2, 6, 1, 17, and 2, respectively. The top three downregulated genes (CCL2, SERPINA3,RARRES1) are located on chromosomes 17, 14,and 3, respectively.

GO enrichment and KEGG pathway analyses were performed to further elucidate the potential biological function and the promising signaling pathways involving the entire 117 DEGs. With GO function analysis, we discovered that the DEGs are mostly enriched in biological process (BP), including the regulation of inflammatory response, the humoral immune response, the regulation of blood pressure, keratan sulfate metabolic process, and the striated muscle cell development. With regard to CC, the DEGs are enriched in the collagen–containing extracellular matrix, vacuolar lumen, blood microparticle, lysosomal lumen. As for molecular function, extracellular matrix structural constituent, signaling receptor activator activity, collagen binding and RAGE receptor binding (Figure 2D). In the KEGG pathway analysis, the DEGs participated in diverse infected signaling pathways, including Malaria, African trypanosomiasis, and Viral protein interaction with cytokine and cytokine receptor, Chagas disease and Legionellosis, and some immune-associated pathways such as the IL-17 signaling pathway (Figure 2E).

### Gene Set Enrichment Analysis

**Figure 3:**
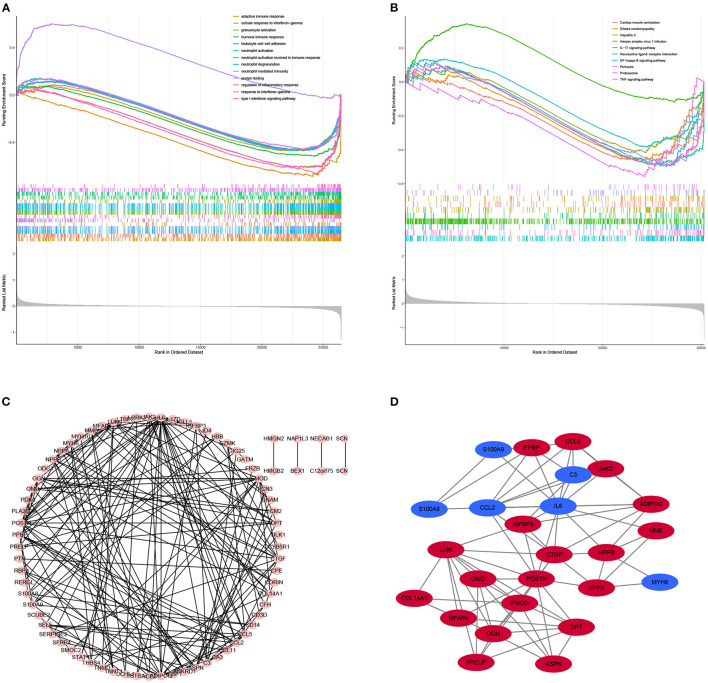
Results of Gene Set Enrichment Analysis (GSEA) plots showing the most enriched gene sets of all detected genes in the DCM cardiac tissues and normal cardiac tissues in an integrated analysis of four datasets. **(A)** The top three most significant up-regulated enriched gene sets in the biological processes: protein folding, humoral immune response, regulation of inflammatory response. **(B)** The top three most significant up-regulated enriched gene sets in the Kyoto Encyclopedia of Genes and Genomes analysis: Herpes simplex virus 1 infection, Neuroactive ligand–receptor interaction, IL−17 signaling pathway. **(C)** PPI network of differentially expressed genes (DEGs). **(D)** Hub gene of differentially expressed genes.

Gene set enrichment analysis was also used to revealed the potential molecular mechanisms of DCM based on all gene information in the gene expression matrix. The enrichment analysis of gene sets revealed that compared to control samples, immune and inflammatory biological processes such as adaptive immune response, cellular response to interferon–gamma, neutrophil activation, protein folding, regulation of inflammatory response are significantly enriched in DCM (Figure 3A). The enriched KEGG pathways of GSEA showed that cardiac muscle contraction, dilated cardiomyopathy, hepatitis C, herpes simplex virus 1 infection and TNF signaling pathway are significantly enriched in DCM (Figure 3B).

### PPI Network Analysis and Screening the Top 25 Genes With Highest Connectivity

**Figure 4:**
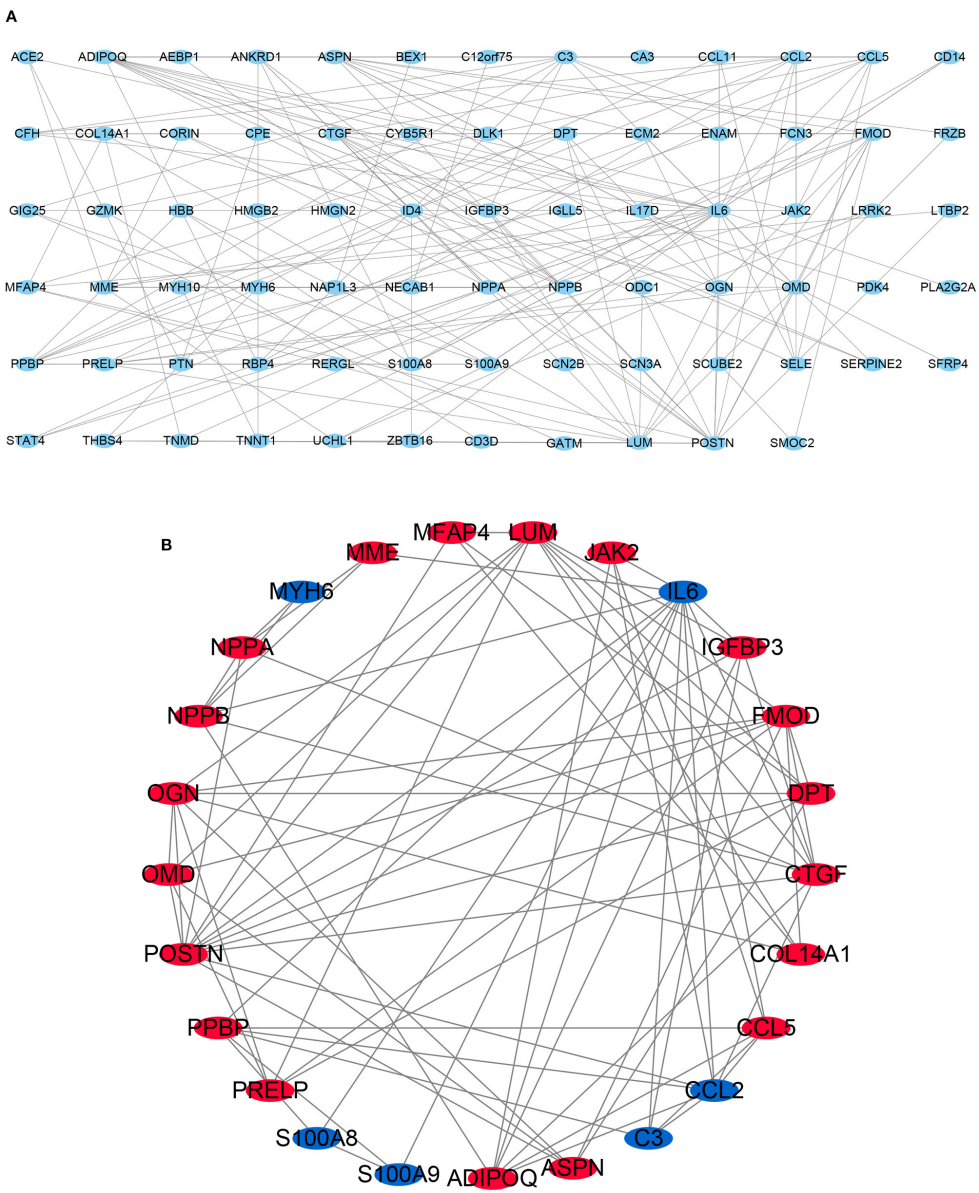
Protein-protein interaction (PPI) network. **(A)** PPI network of 117 differentially expressed genes (DEGs). **(B)** subnetwork of top 25 genes with the highest degree of connectivity from the PPI network. Node color reflects the degree of connectivity (Red color represents a higher expression, and blue color represents a lower expression).

STRING is an online tool for investigating and integrating interaction between proteins (21). PPI network of these genes was obtained after imputing the DEGs into the online tool STRING (Figures 3C, 4A). In order to identify the top highest connectivity 25 genes in the network, the PPI network was imported into Cytoscape. And then, the top 25 genes with the highest degree of connectivity were calculated and extracted. Subsequently, the top 25 genes with the highest degree of connectivity were inputted into STRING to detect the interaction between proteins encoded by these genes (Figures 3D, 4B).

### Validation of the Expression of the Top 25 Genes With Highest Connectivity in Independent Patient Cohorts

**Figure 5:**
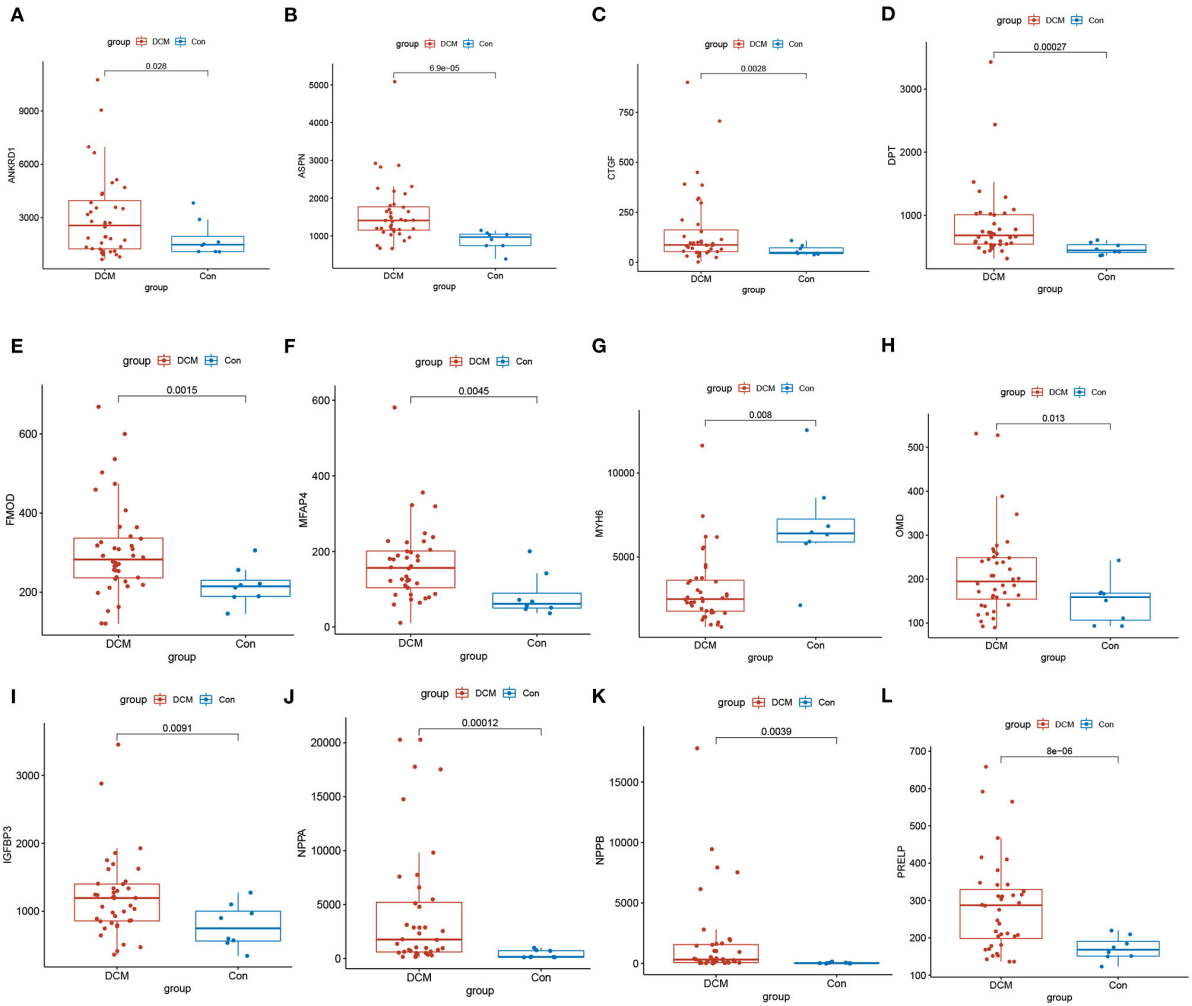
Validation of top 25 genes with the highest degree of connectivity in GSE17800. The expression of genes **(A)** ANKRD1, **(B)** ASPN, **(C)** CTGF, **(D)** DPT, **(E)** FMOD1, **(F)** MFAP4, **(H)** OMD, **(I)** IGFBP3, **(J)** NPPA, **(K)** NPPB, **(L)** PRELP significantly upregulated, and **(G)** MYH6 significantly down regulated in the DCM in comparison to control group. DCM, dilated cardiomyopathy; Con, control group.

There are 48 cardiac tissue samples including 40 DCM tissues and eight control tissues in GSE17800 dataset. This microarray also reported the information of patient's clinical features (Table 2). Independent patient cohorts fron GSE17800 dataset was used to verify the top 25 genes mRNA levels in DCM, which indicated that expression of ANKRD1, ASPN, CTGF, DPT, FMOD, MFAP4, OMD, JAK2, NPPA, NPPB and IGFBP3 was also significantly up-regulated and MYH6 was significantly down-regulated in DCM cardiac tissues as compared to normal cardiac tissues (Figure 5).

**Table 2:** The clinical characteristics of GSE17800 in dilated cardiomyopathy.

**Parameter**	**DCM group** **(***N*** = 40)**	**Control group** **(***N*** = 8)**	***p*** **value**
Age, years	50.21 ± 9.35	43.13 ± 14.76	0.084
Gender (Male/Female)	28/12	6/2	0.887
BMI, kg/m^2^	27.88 ± 4.57	26.33 ± 5.26	0.303
LVEF, %	33.3 ± 6.4	59.8 ± 8.0	<0.001
LVIDD, mm	69.8 ± 8.0	51.4 ± 3.1	<0.001
Virus(Positive/Negative)	22/18	0/8	0.000
Inflammation	22 ± 23 or 18(12–22)	10 ± 3 or 10(8–13)	<0.001

*BMI, body mass index; LVEF, left ventricular ejection fraction; LVIDD, left ventricular internal diameter at end-diastole*.

### The Identification of Hub Genes and Functional Annotation Analysis

**Figure 6:**
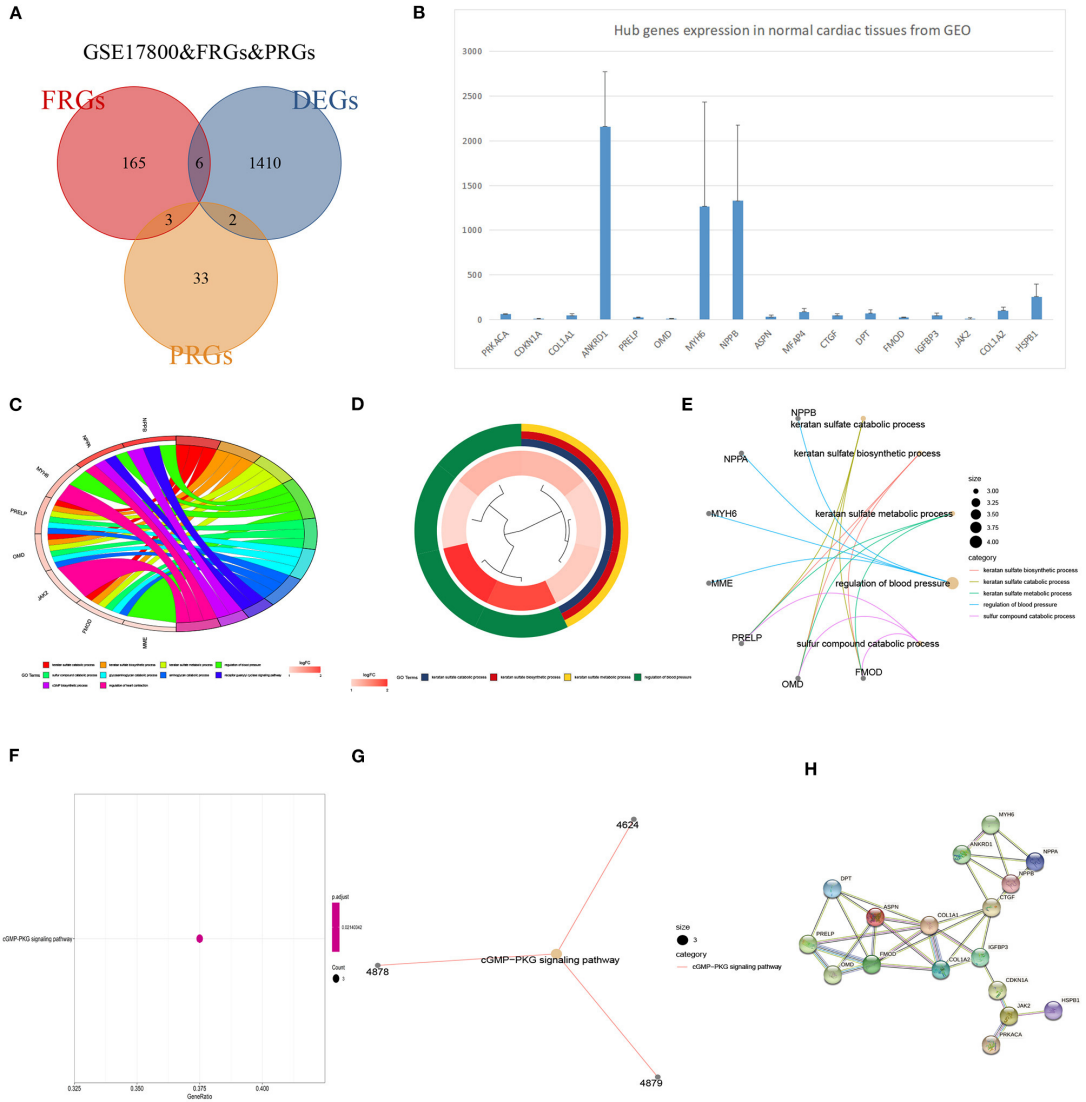
The identification of DCM related genes and their functional analysis. **(A)** Venn diagram of ferroptosis-related genes, pyroptosis-related genes and DEGs in GSE17800. **(B)** The expression of potential biomarker genes including top 25 genes validated in GSE17800 and overlapping DEGs of ferroptosis-related genes, pyroptosis-related genes based on NCBI. **(C)** Chord plot shows the distribution of core genes in different GO-enriched functions. Symbols of core genes are presented on the left side of the graph with their fold change values mapped by color scale. **(D)** The top three significantly enriched Gene Ontology terms associated with potential biomarker genes. Gene involvement in the GO terms was determined by colored connecting lines. **(E)** Cluego network diagram shows the relationship between the potential biomarker genes and GO terms. **(F)** The bubble chart showed the KEGG pathway analyses of potential biomarker genes in DCM. **(G)** Cluego network diagram shows the relationship between the potential biomarker genes and KEGG terms. **(H)** PPI network of 18 potential biomarker genes.

GSE17800 was also processed as previous. DEGs between DCM and normal cardiac tissues in GSE17800 were 1410. To explore potential ferroptosis or pyroptosis related genes in dilated cardiomyopathy, we intersected FRGs and PRGs with GSE17800's DEGS, and obtained 6 ferroptosis related differential genes including YY1AP1, CDKN1A, SRC, SESN2, CBS, and HSPB1 and 2 pyroptosis related differential genes including PRKACA and COL1A1 (Figure 6A). Next, the 13 high connectivity genes after validation and 8 FRGs/PRGs above were further import into NCBI respectively to test their expressive abundance in normal cardiac tissues. These genes with over than 5 mean RPKM were regarded as the hub genes (Figure 6B). To reveal potential biological process of these hub genes, GO and KEGG analyses were conducted. The most significant GO terms for biological process, keratan sulfate catabolic process, and keratan sulfate biosynthetic process, as well as KEGG pathways, were shown in Figures 6C–G. These analysis showed that these hub genes were mainly involved in keratan sulfate process, heart process and cGMP metabolic process. The PPI network of the 18 hub genes was also performed, which showed an interaction among them (except for YY1AP1) (Figure 6H).

### Several Hub Genes Play a Diagnostic Role in DCM

**Figure 7:**
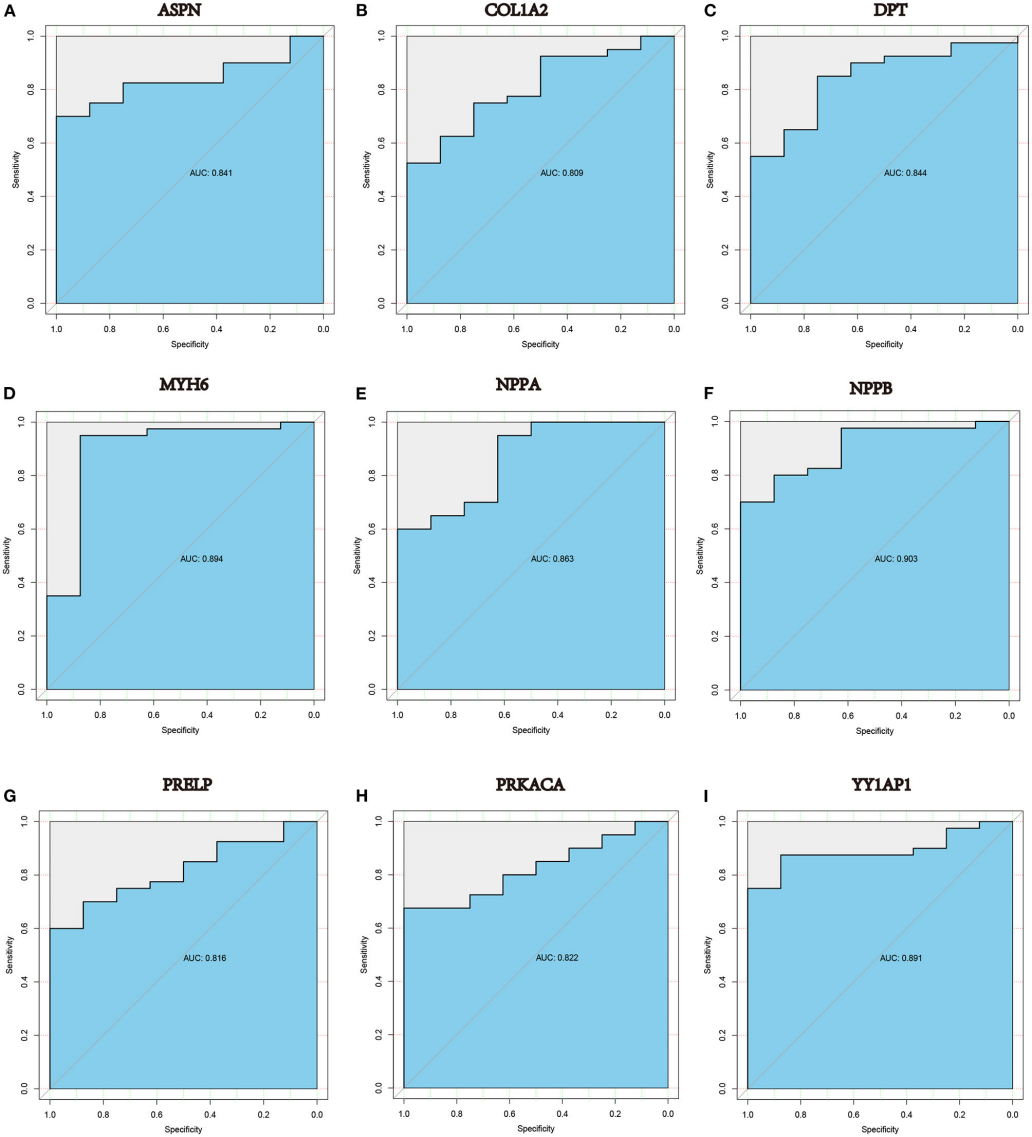
ROC curve analysis in GSE17800 demonstrated that **(A)** ASPN, **(B)** COL1A2, **(C)** DPT, **(D)** MYH6, **(E)** NPPA, **(F)** NPPB, **(G)** PRELP, **(H)** PRKACA and **(I)** YY1AP1 may be diagnostic biomarkers in patients with DCM. AUC: area under the curve.

A ROC curve analysis was performed to evaluated the diagnostic value of these hub genes in DCM. The results indicated that many genes, including ASPN (*AUC* = 0.841, *P* < 0.0001), COL1A2 (*AUC* = 0.809, *P* < 0.0001), DPT (*AUC* = 0.844, *P* < 0.0001), MYH6 (*AUC* = 0.894, *P* < 0.0001),NPPA (*AUC* = 0.863, *P* < 0.0001), NPPB (*AUC* = 0.903, *P* < 0.0001), PRELP (*AUC* = 0.816, *P* < 0.0001), PRKACA (*AUC* = 0.822, *P* < 0.0001) and YY1AP1 (*AUC* = 0.891, *P* < 0.0001), can efficiently distinguish DCM cardiac tissues from normal cardiac tissues (Figures 7A–I).

### LAASO Model for Predicting DCM and Correlation of Clinicopathological Features and Model Genes

**Figure 8:**
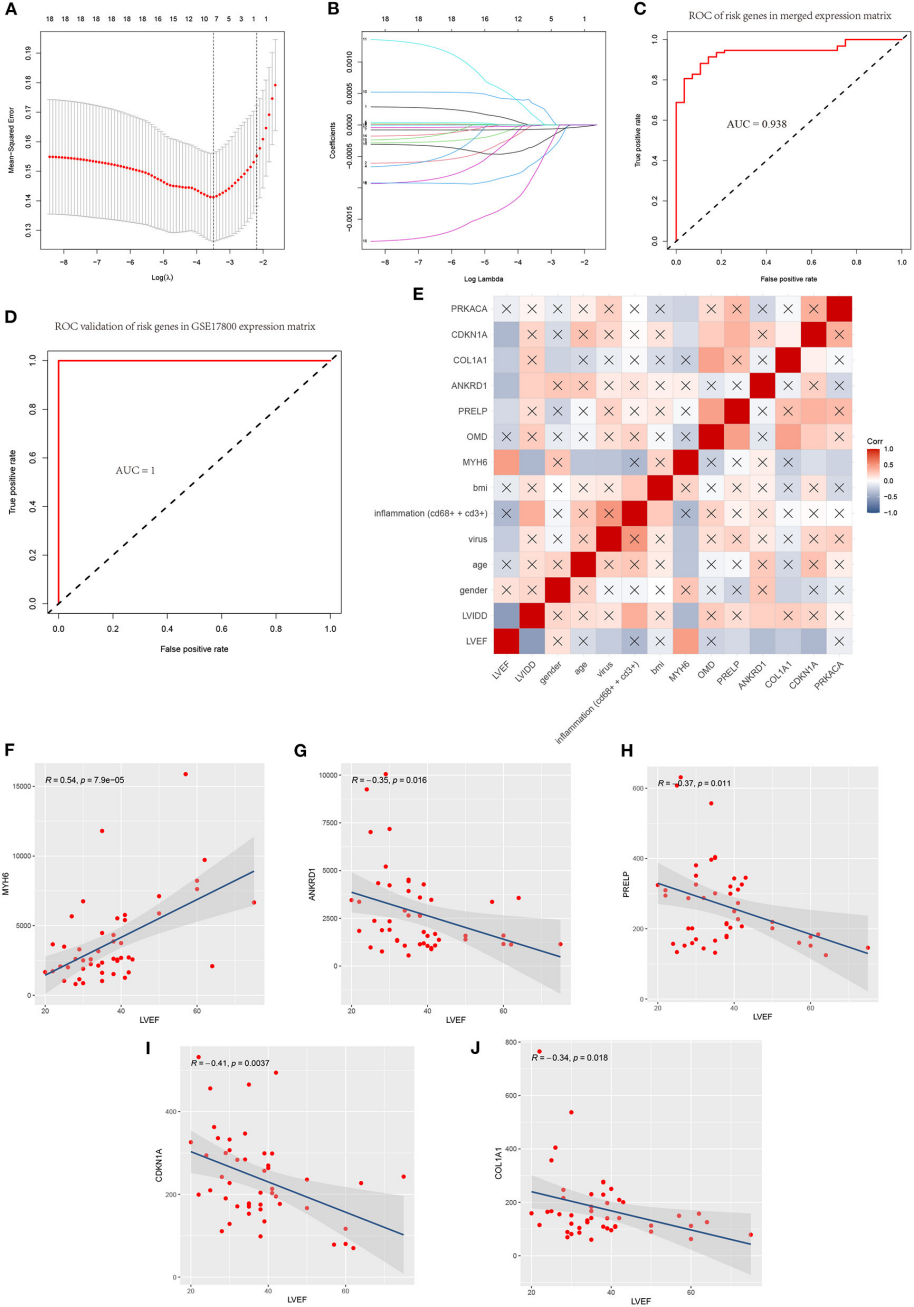
A model for predicting DCM and correlation of clinicopathological features and model genes. **(A)** Least absolute shrinkage and selection operator (LASSO) logistic regression algorithm to screen diagnostic markers and risks genes in merged data matrix of five datasets. **(B)** Parameters of Lasso path and corresponding selected features of each fold. **(C)** ROC curve analysis of the 7-gene-based model in merged data matrix of five datasets (training set). **(D)** ROC curve analysis of the 7-gene-based model in GSE17800(validation set). **(E)** Correlation heat map of risks genes and clinicopathological features in datasets GSE17800. The depth of the color represents the strength of the correlation; red represents a positive correlation, blue represents a negative correlation. The “x” means irrelevance. Correlation analysis of left ventricular ejection fraction (LVEF) and **(F)** MYH6, **(G)** ANKRD1, **(H)** PRELP, **(I)** CDKN1A and **(J)** COL1A1.

We extracted the expression profile of 18 hub genes from the merged datasets to construct LASSO model (Figures 8A,B). Through the LASSO, 7 genes were identified with non-zero regression coefficients, and the value of lambda.min = 0.03037093. ROC curve analysis indicated that the AUC of the 7-gene-based model was 0.938 in the merged gene set, which suggesting LASSO model may be used as a biomarker of DCM (Figure 8C). This model was further validated in a validation set (GSE17800) with AUC= 1 (Figure 8D).

Correlation heatmap of the 7 model genes and clinical factors revealed that LVEF had a significant positive correlation with MYH6 and had a significant negative correlation with LVIDD, ANKRD1, PRELP, COL1A1, CDKN1A. LVIDD had a significant negative correlation with MYH6 and had a significant positive correlation with inflammation, ANKRD1. Age and MYH6 had a significant negative correlation. The expression of MYH6 mRNA levels is associated with virus infection (Figures 8E–J).

### Identification of the Potential Drugs

**Figure 9:**
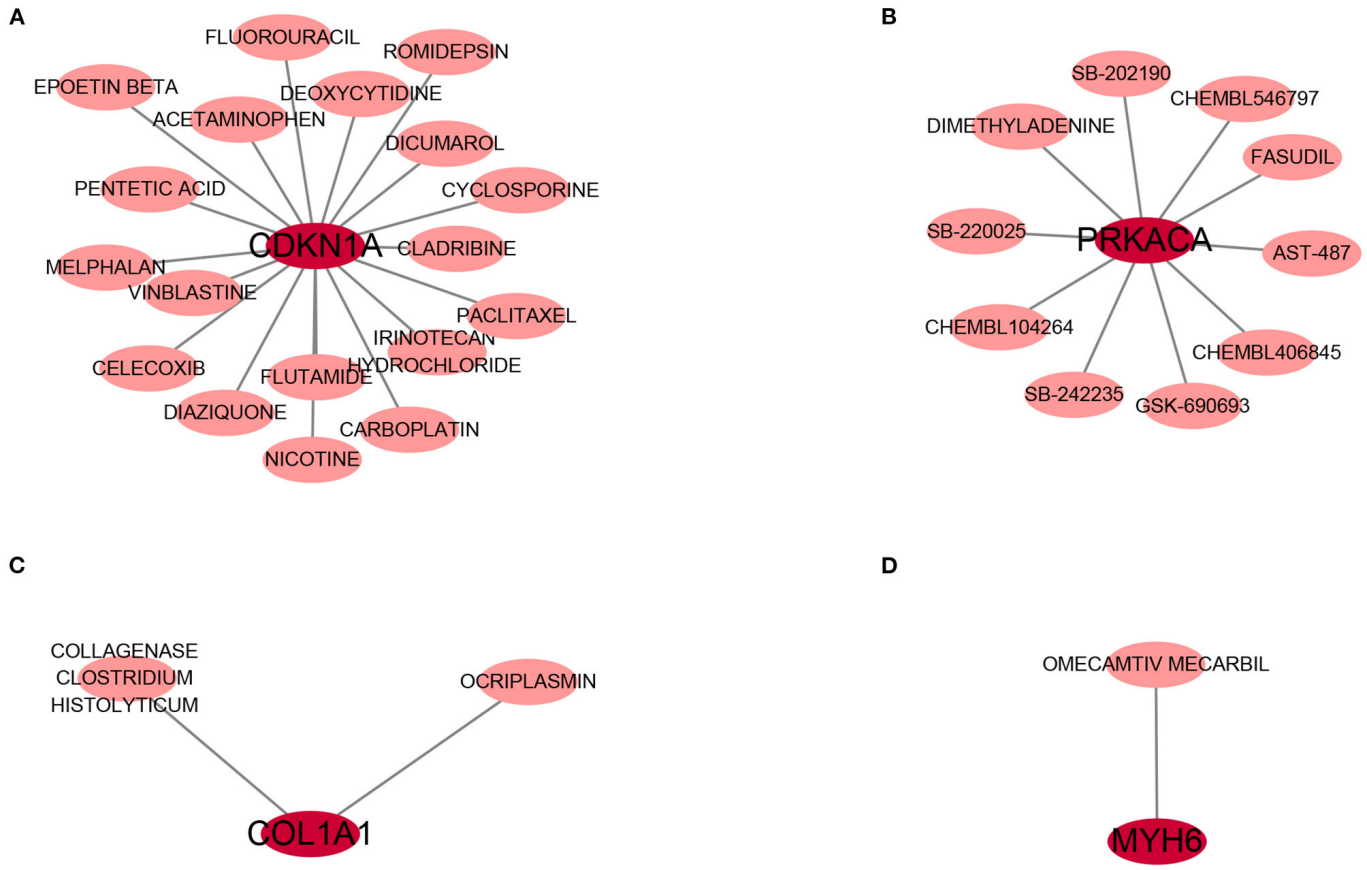
The drug-gene interaction network. The dark red circle nodes in the center are the genes, and pale red nodes around are the drugs. **(A)** The interaction network of CDKN1A and its targeted compounds and drugs. **(B)** The interaction network of PRKACA and its targeted compounds and drugs. **(C)** The interaction network of COL1A1 and its targeted compounds and drugs. **(D)** The interaction network of MYH6 and its targeted compounds and drugs.

DGIdb was applied to determine the potential therapy drug that could reverse the expression of model gene in DCM. As shown in the drug–gene interaction network (Figures 9A–D), 18 drugs or molecular compounds included deoxycytidine, irinotecan hydrochloride, and cyclosporine, which differentially regulated the expression of ferroptosis-related gene CDKN1A. In addition, OMECAMTIV MECARBIL (INN), a cardiac specific myosin activator, was found to interact with MYH6. Further, collagenase clostridium histolyticum and antiplasmin regulated COL1A1 and 10 drugs or molecular compounds that included fasudil, SB-220025, SB-202190 and AST-487 regulated pyroptosis related gene PRKACA.

## Discussion

The pathogenesis of DCM, a complex and heterogeneous disease, remains unclear (28). Although many investigators have used microarray and RNA-seq to detect novel biomarkers and therapeutic targets for DCM, inconsistencies were seen between the DEGs found in different studies. To our knowledge, our work is the first to use RRA combined with LASSO regression model to explore novel hub genes associated with DCM. Previous studies compared gene expression profiles between DCM and non-heart failure samples for the dataset to explore hub gene and pathogenesis in DCM. However, these studies did not conduct link between gene expression and DCM clinical characteristics. This study integrated 4 qualified DCM datasets from GEO into the RRA method to identify DCM-associated genes and develop expression-based molecular signatures to predict DCM, some of which, such as NPPB (29) and ASPN (30), have been reported to be biomarkers of DCM or play an important role in its pathogenesis. In addition, associations of developed signatures with the clinicopathological characteristics of DCM were also evaluated.

Different from previous studies, GO analysis of differential genes in this study mainly involves the regulation of inflammatory response, immune response, keratinin sulfate processing and muscle cell contraction. And KEGG pathway analysis is mainly involved diverse infected signaling pathways, including Malaria, African trypanosomiasis, and Viral protein interaction with cytokine and cytokine receptor, Chagas disease and Legionellosis, and some immune-associated pathways such as the IL-17 signaling pathway. In addition, GSEA clarified a new perspective for this study. It demonstrated that immune responses, such as adaptive immune response, cellular response to interferon–gamma, type I interferon signaling pathway, and inflammation responses including leukocyte cell-cell adhesion, neutrophil activation, neutrophil degranulation are involved in the pathophysiological process of dilated cardiomyopathy. Also, DCM process, some kinds of infections and two immune-related disease pathways that include IL-17 signaling pathway and TNF signaling pathway were involved. These confirmed that infection, inflammation and immune responses play important role in the development DCM.

Ferroptosis is reliant on a large number of cellular iron, interfering with the homeostasis of redox reactions, and eventually promoting cell death (31). Literature has indicated that iron-dependent ferroptosis is implicated in many cardiomyopathies (32). However, no studies have shown that ferroptosis is involved in the development of dilated cardiomyopathy; Moreover, pyroptosis is also another form of cell death, which has only been shown in one study that NLRP3 inflammasome-mediated pyroptosis contributes to the pathogenesis of non-ischemic dilated cardiomyopathy (33). Therefore, the present study also for the first time reported ferroptosis related gene CDKN1A and pyroptosis related gene PRKACA may be involved in the development and progression of dilated cardiomyopathy.

We preliminarily obtained the possible core genes through protein interaction network and iron death or cell apoptosis related differential gene screening, and then carried out independent data set verification and expression abundance verification, and finally obtained the included 18 core genes. The 18 genes included up-regulated genes CDKN1A, COL1A1, ANKRD1, PRELP, OMD, NPPB, NPPA, ASPN, MFAP4, CTGF, DPT, FMOD, IGFBP3, JAK2, PRKACA and COL1A2, as well as down-regulated genes MYH6 and YY1AP1, which were mainly enriched in keratan sulfate process and cGMP–PKG signaling pathway through GO and KEGG analyses. some of them were demonstrated to exert essential roles in the pathogenesis of DCM (34–37). Among these hub genes, we further explore their diagnostic value in DCM, and found that nine genes have certain diagnostic value for dilated cardiomyopathy (AUC > 80%). Among them, NPPA (natriuretic peptide A) and NPPB (natriuretic peptide B) belong to the natriuretic peptide family, which encoded atrial natriuretic peptide (ANP) and brain natriuretic peptide (BNP) respectively (36). The natriuretic peptide family is a general name of a group of peptides secreted mainly by the cardiovascular system to regulate hydroelectrolyte balance, reduce cardiac afterload, and dilate blood vessels through natriuretic diuresis (38). In heart failure, NPPB is expressed at a high level in DCM, and patients with higher BNP level have a worse cardiac function (39, 40).

Combined LASSO and the forward stepwise selection analyses of the 18 genes resulted in the most robust model with the fewest genes capable of predicting DCM, a panel of seven genes including ANKRD1, PRELP, PRKACA, COL1A1, OMD, MYH6 and CDKN1A significantly correlates with DCM. This seven-gene panel was named DCM derived diagnostic signature (DCMDDS), and the DCMDDS score is based on the seven genes' expression levels and regression coefficients. The model was validated in 5 patient cohorts, comprising more than 120 patients. Interestingly, five of the seven DCMDDS genes were associated with left ventricular ejection fraction based on their expression levels and correlation coefficients, as demonstrated by the Spearman correlation analysis. Among DCMDDS genes, MYH6, ANKRD1 and COL1A1 have been showed to participated in the development of DCM, while PRELP, PRKACA, CDKN1A and OMD seldomly reported.

PERELP, proline and arginine rich end leucine rich repeat protein, is a member of the leucine-rich repeat (LRR) family of extracellular matrix proteins in connective tissue (41). It is unclear whether PERELP plays a role in DCM and other cardiomyopathy. Data from the Human Protein Atlas (HPA) revealed that PRELP is secreted to the extracellular matrix and may anchor basement membranes to the underlying connective tissue (42), suggesting its potential function in maintaining normal cellular structure. Besides, previous studies have suggested that PERELP has prognostic value in hepatocellular carcinoma and regulates the extracellular matrix and collagen mineralization in the bone system (43, 44).

PRKACA is a gene encoding the cAMP-dependent protein kinase A (PKA) catalytic subunits alpha. PKA can directly phosphorylate the cytoplasmic receptor NLRP3 and attenuate its ATPase function, which showed a relationship to pyroptosis (45). Therefore, PRKACA was regarded as a pyroptosis-related gene (24). Prolonged and elevated cyclic adenylyl monophosphate (cAMP) levels have been observed in both heart failure and several cardiomyopathy, while PKA is promptly activated by increasing intracellular concentrations of cAMP synthesized by adenylyl Cyclases (46, 47). These revealed that regulating PKA phosphorylation may be a therapeutic strategy for certain stages of progressive and congestive heart failure (48). However, its role in DCM still not be reported.

CDKN1A (cyclin-dependent kinase inhibitor 1A) encodes p21, plays an important role in the pathological process of P53-mediated ferroptosis (49). CDKN1A is a potent cell cycle inhibitor that mediates post-natal cardiomyocyte cell cycle arrest. Although no reported in DCM, CDKN1A is implicated in LMNA-mediated cellular stress responses, and the mutation of LMNA is one of the important mechanisms DCM (50, 51). Moreover, Shah et al. also found the mutations in the CDKN1A gene in the blood of patients with heart failure (52).

OMD (Osteomodulin) is a leucine- and aspartic acid-rich keratan sulfate proteoglycan, which belongs to the small leucine-rich proteoglycan family (SLRP) family (53). A recent study shown that OMD could directly bind to Type I collagen, further regulating the diameter and shape of collagen fbrils (54). Interestingly, the results of the present study also suggest a positive correlation between OMD and COL1A1, and its functional analysis mainly involved in extracellular matrix processes. Extracellular matrix fibrosis is regarded as an important process in the development of DCM (55). Therefore, targeted OMD gene therapy may be a potential therapeutic strategy for dilated cardiomyopathy. Guo et al. also found that Osteomodulin is a potential genetic target for hypertrophic cardiomyopathy (56).

To found the potential effective therapy for DCM, DGIdb database was used to exam therapeutic agents that might reverse the abnormally expression of DCMDDS genes. OMECAMTIV MECARBIL (INN), previously codenamed CK-1827452, is a cardiac specific myosin activator. It is clinically tested for its role in the treatment of left ventricular systolic heart failure (57). In the present study, MYH6 was down-regulated in DCM, while OMECAMTIV MECARBIL can target and activate it. This means that MYH6 may be one of the important targeted gene of OMECAMTIV MECARBIL in the treatment of heart failure. COL1A1, one of the component collagen type I, is the main component of extracellular matrix. Elevated expression levels of COL1A1 will lead to fibrosis of extracellular matrix of cardiac muscle (58). Collagenase clostridium histolyticum and antiplasmin might be a effective anti-fibrosis therapy approach in DCM via targeting COL1A1. The roles of the drugs or molecular compounds above in DCM still need to be further explored as potential therapeutic targets.

There were several limitations in our study. First, due to limited conditions, myocardial biopsy tissue specimens were not obtained to carry out basic experiments for verification. Nevertheless, we used a multi-chip combined analysis method and validated in external DCM samples to ensure the accuracy of the bioinformatics analysis in the study. In addition, in our analysis results, NPPA, NPPB, COL1A2, ASPN, ANKRD1 and CTGF were all confirmed to be closely related to heart failure in dilated cardiomyopathy in various studies. Second, the sample size of our multi-chip combined analysis was significantly expanded. However, due to the difficulty and high risk of myocardial tissue biopsy, the sample size of the data set in our study was still relatively small. Third, although we explored the relationship between genes and clinical factors, we failed to obtain prognostic information from datasets, and we could not further explore the relationship between DCMDDS genes and patient outcomes.

In summary, by combining RRA, LASSO, and other bioinformatics tools, this study identified 117 robust DEGs between DCM and NFH samples, many of which were not reported in previous studies. A 7-gene panel derived from the 117 DCM-associated genes comprised of a diagnostic model predictive of DCM. Five of the seven genes were closely related to left ventricular ejection fraction. Therefore, these gene signatures may help develop DCM biomarkers via large-scale randomized clinical trials.

## Data Availability Statement

The datasets presented in this study can be found in online repositories. The names of the repository/repositories and accession number(s) can be found in the article/supplementary material.

## Author Contributions

XM, CM, and CG designed the present study, which was performed by XM, CM, and PC. XM and LH made substantial contributions to acquisition and analysis of data. XM and LS also made contributions to interpretation of data. XM wrote the initial draft of the manuscript. LH revised it critically for important intellectual content. All authors have participated sufficiently in the work to take public responsibility for appropriate portions of the content and approved the manuscript, as well as agreed to be accountable for all aspects of the work. All authors read and approved the final manuscript.

## Funding

The present study was supported by the National Nature Science Foundation of China (Grant No. 81770333).

## Conflict of Interest

The authors declare that the research was conducted in the absence of any commercial or financial relationships that could be construed as a potential conflict of interest.

## Publisher's Note

All claims expressed in this article are solely those of the authors and do not necessarily represent those of their affiliated organizations, or those of the publisher, the editors and the reviewers. Any product that may be evaluated in this article, or claim that may be made by its manufacturer, is not guaranteed or endorsed by the publisher.
