# Corrigendum: Robust rank aggregation and least absolute shrinkage and selection operator analysis of novel gene signatures in dilated cardiomyopathy

**DOI:** 10.3389/fcvm.2022.1002803

**Published:** 2022-10-14

**Authors:** Xiao Ma, Changhua Mo, Liangzhao Huang, Peidong Cao, Louyi Shen, Chun Gui

**Affiliations:** Department of Cardiology, First Affiliated Hospital of Guangxi Medical University, Nanning, China

**Keywords:** dilated cardiomyopathy, Robust Rank Aggregation, novel gene signatures, Least absolute shrinkage and selection operator analysis, PERELP

In the published article, there was an error in the article title. Instead of “An Robust Rank Aggregation and Least Absolute Shrinkage and Selection Operator Analysis of Novel Gene Signatures in Dilated Cardiomyopathy,” it should be “Robust Rank Aggregation and Least Absolute Shrinkage and Selection Operator Analysis of Novel Gene Signatures in Dilated Cardiomyopathy.”

In the published article, due to the previous corresponding author's email address becoming unavailable, it has been updated. Instead of “guichun@yahoo.com,” it should be “gui_chun@163.com.”

The authors apologize for this error and state that this does not change the scientific conclusions of the article in any way. The original article has been updated.

